# Minimally Invasive Pancreatoduodenectomy Compared to Open Pancreatoduodenectomy in Patients With Pancreatic Cancer: A Systematic Review

**DOI:** 10.7759/cureus.89264

**Published:** 2025-08-02

**Authors:** Mariana Olvera Morales, Diego Santillán Alcántar, Jorge Marín Ramírez, Jorge A Romero Chávez, Jaime Alvarez-Gutierrez, Jesús R Ventolero Carbajal, Joaquín Raya Moreno, Jorge L Jiménez Rosas, José C Hernández González, Jennifer P Paxtor Quetuc, Graciela G Andrade Váquiz, Jose R Flores Valdés

**Affiliations:** 1 General Medicine, Universidad Autónoma de Guadalajara, Guadalajara, MEX; 2 General Surgery, Instituto Mexicano del Seguro Social Regional General Hospital No. 46, Guadalajara, MEX; 3 General Medicine, Universidad Autónoma de Guadalajara, Villahermosa, MEX; 4 General Medicine, ⁠⁠Universidad Autónoma de Durango Campus Zacatecas, Zacatecas, MEX; 5 General Surgery, Regional Hospital “Dr. Valentin Gomez Farias”, Institute of Security and Social Services for the State Workers, Zapopan, MEX; 6 General Medicine, Universidad Autónoma del Estado de México, Ciudad de México, MEX; 7 General Medicine, ⁠⁠Instituto Tecnológico de Estudios Superiores Monterrey, Monterrey, MEX; 8 General Medicine, Universidad Anáhuac México, Ciudad de México, MEX; 9 General Medicine, Universidad Nacional Autónoma de México, Ciudad de México, MEX; 10 General Medicine, ⁠⁠Universidad de San Carlos de Guatemala, Santa Elena, GTM; 11 General Medicine, Universidad de El Salvador, San Salvador, SLV; 12 General Physician, Oncology Consultants, Houston, USA

**Keywords:** minimally invasive, open surgery, pancreatic cancer, pancreatic resection, pancreatoduodenectomy

## Abstract

Pancreatic ductal adenocarcinoma is one of the most lethal malignancies, with a five-year survival rate below 8%. For resectable cases, pancreatoduodenectomy remains the standard treatment. While the open approach has traditionally been the primary treatment, minimally invasive techniques are gaining popularity due to advancements in surgical technology. This review evaluates postoperative safety and long-term outcomes of minimally invasive pancreatoduodenectomy compared to open pancreatoduodenectomy in patients with pancreatic ductal adenocarcinoma.

A comprehensive database search yielded 636 articles, of which 9 studies met our inclusion criteria and were retrieved for analysis. These comprised eight cohort studies and one randomized controlled trial, including a total of 13,159 participants.

Findings suggest that minimally invasive pancreatoduodenectomy may offer benefits, such as reduced blood loss, shorter hospital stays, faster recovery, and comparable surgical outcomes, making it a promising alternative to open pancreatoduodenectomy. However, minimally invasive approaches are also associated with challenges such as longer operative times and increased technical complexity. Therefore, further research and structured surgical training are essential to optimize clinical outcomes and guide decision-making.

## Introduction and background

The pancreas is a retroperitoneal organ in the human body with both exocrine and endocrine functions. Pancreatic tumors can be classified into two groups: Non-endocrine pancreatic tumors and endocrine pancreatic tumors. Non-endocrine pancreatic tumors can be benign or malignant. Adenoma, cystadenoma, lipoma, fibroma, hemangioma, lymphangioma, and neuroma are benign non-endocrine pancreatic tumors. Malignant pancreatic tumors display a range of histological characteristics, including ductal adenocarcinoma, sarcoma, and metastatic tumors [1]. Approximately 90% of pancreatic cancers are pancreatic ductal adenocarcinoma (PDAC) [2]. Patients diagnosed with this disease typically have a median survival of less than six months, and only about 8% exhibit a survival rate of five years. Patients often remain asymptomatic until the cancer has disseminated throughout the body. Likewise, treatments for pancreatic cancer are relatively ineffective, failing to extend patients' life expectancy by more than a few months [3].

Currently, pancreatic cancer is the third leading cause of cancer-related deaths in the United States and is expected to become the second leading cause by 2030. In 2020, about 500,000 new cases were registered, and 470,000 deaths were attributed to pancreatic cancer across the world. In the same year, the global incidence rate for pancreatic cancer was 4.9 per 100,000 for both sexes combined, while the mortality rate was 4.5 per 100,000 [4]. Pancreatic cancer tends to affect men at a higher rate than women [5]. Some of the risk factors include age, sex, family history, genetic susceptibility, diabetes, pancreatitis, obesity, smoking, and alcohol consumption, among others [6].

Pancreaticoduodenectomy is a complex surgery and remains the standard treatment for various pathologies affecting the pancreatic head, duodenum, ampulla, and distal bile duct, such as pancreatic and periampullary tumors. Despite significant advances over the past decade, pancreatoduodenectomy continues to be associated with substantial perioperative morbidity that impacts the patient’s quality of life. The traditional approach for this surgical procedure involves open surgery. Nevertheless, in the last decades, open surgery has been replaced by minimally invasive surgery to reduce surgical trauma, aiming for better postoperative recovery and results [7,8]. Minimally invasive surgery, introduced in the 1980s, has transformed the field of surgery by reducing morbidity. It has also gained support from the 2019 Miami evidence-based guidelines, with studies demonstrating reduced blood loss and quicker functional recovery compared to open procedures. Minimally invasive surgery can be carried out using laparoscopic or robotic techniques. The first reported laparoscopic pancreatoduodenectomy (LPD) case in 1994 indicated shorter hospital stays and fewer complications in some studies. However, other studies have reported higher morbidity and mortality rates associated with the technique. On the other hand, the learning curve for LPD is quite long and can only be overcome in high-volume centers. This is not the case with robotic pancreaticoduodenectomy (RPD), which has a shorter learning curve [7-9].

Despite numerous studies comparing the treatment outcomes of minimally invasive pancreaticoduodenectomy (MIPD) with open pancreaticoduodenectomy (OPD), there is still a lack of information on the long-term outcomes of both techniques, especially in patients diagnosed with pancreatic cancer. Some areas have not received enough emphasis, and there is a lack of available information [10,11]. Therefore, evaluating the postoperative complications these procedures may pose for patients is crucial. For example, how do these procedures impact the patient’s quality of life, survival, and long-term pancreatic function? Is there a significant difference between the risks and benefits of such operations? This systematic review aims to compare the safety and long-term outcomes of MIPD versus OPD specifically in patients diagnosed with PDAC.

## Review

Methods

Search Strategy

The systematic review adheres to the standards established by the Preferred Reporting Items for Systematic Reviews and Meta-Analysis (PRISMA 2020), while also adhering to the methodological guidance provided in 10 Steps to Conduct a Systematic Review by Calderon Martinez et al. [12,13]. Both resources are freely accessible under the Creative Commons Attribution license. With registration officially obtained and validated through PROSPERO with the ID CRD42025610006.

We conducted a systematic review of the literature to assess the postoperative outcomes of MIPD in comparison to OPD. We access various databases, including PubMed, Cochrane, ScienceDirect, and Medical Subject Headings (MeSH) terms covering the period from January 2003 to September 2024. The key search terms employed included “pancreatoduodenectomy”, “pancreatic cancer”, “pancreatic resection”, “minimally invasive”, and “open surgery”.

Type of Study

We performed a systematic review of relevant studies published between 2012 and 2023, which are available in English. These studies were required to compare treatment outcomes and major complications between MIPD and OPD. The studies that met our inclusion criteria were randomized clinical trials (RCTs) and cohort studies.

Exclusions were applied to case reports, dissertations, book chapters, news articles, conference abstracts, commentary publications, and letters to the editor. Additionally, we excluded duplicate studies, those lacking a detailed methodology, and studies for which we could not obtain the necessary data or did not receive a response from the author via email.

Type of Participants

This study established specific participant selection criteria, including individuals over 18 years of age diagnosed with PDAC. There were no restrictions regarding sex, ethnicity, place of residence, income, or health insurance status. The study excluded populations such as pediatric patients under 18 years of age, pregnant individuals, and animals.

Type of Intervention

To be eligible for inclusion in this study, the research involved adult patients who were candidates for elective partial pancreatoduodenectomy, whether performed using minimally invasive or open techniques, and had to report at least one postoperative outcome. Studies that compared the outcomes of both procedures were also included.

Articles using other procedures, such as neoadjuvant pancreatic radiotherapy, studies that did not report postoperative outcomes, and studies that involved patients who did not undergo surgical intervention were excluded.

Outcomes

This study evaluates MIPD and OPD, with postoperative long-term outcomes as our primary focus. The analysis included the number of patients with a Clavien-Dindo classification score of 3 or higher, the number of patients with complications, and the mortality rate for each procedure. Among the secondary outcomes, we assessed the number of patients who required a blood transfusion and the duration of the surgery. Outcomes were categorized based on the type of procedure to facilitate a direct comparison between MIPD and OPD.

Selection of Studies

After an initial screening of titles and abstracts, two reviewers (JLJR, AVGG) independently evaluated studies for inclusion in this review according to established inclusion and exclusion criteria. Any discrepancies in the selection process were resolved through consensus and by consulting a third reviewer (JAAG). During the full-text review phase, two reviewers (JLJR, JARC) independently assessed the studies based on the same criteria. Disagreements were resolved through consensus and, if needed, by consulting a third reviewer (MOM).

Assessment of Risk of Bias in Included Studies

The ROB2 tool, which is freely available and open access, was used to assess the risk of bias (ROB) in the RCTs [14]. Two independent reviewers (JRVC, DSA) evaluated the article based on the criteria established by this tool. Any discrepancies between the two reviewers were resolved by a third reviewer (JPPQ), who also conducted the assessment. The results were reported according to the quality of evidence as Low risk, Some concerns, or High risk, depending on the criteria met in each domain.

For the cohort study assessment, we applied the Newcastle-Ottawa Quality Assessment Scale criteria (NOS) [15]. This review was also conducted by two independent reviewers (JRVC, DSA), with any discrepancies resolved by a third reviewer (JPPQ). The results were reported as Good quality, Fair quality, or Poor quality.

Results

We conducted a systematic review of the literature to compare the results of MIPD versus OPD, gathering available information from PubMed, Cochrane, and ScienceDirect from January 2003 to September 2024.

**Figure 1:**
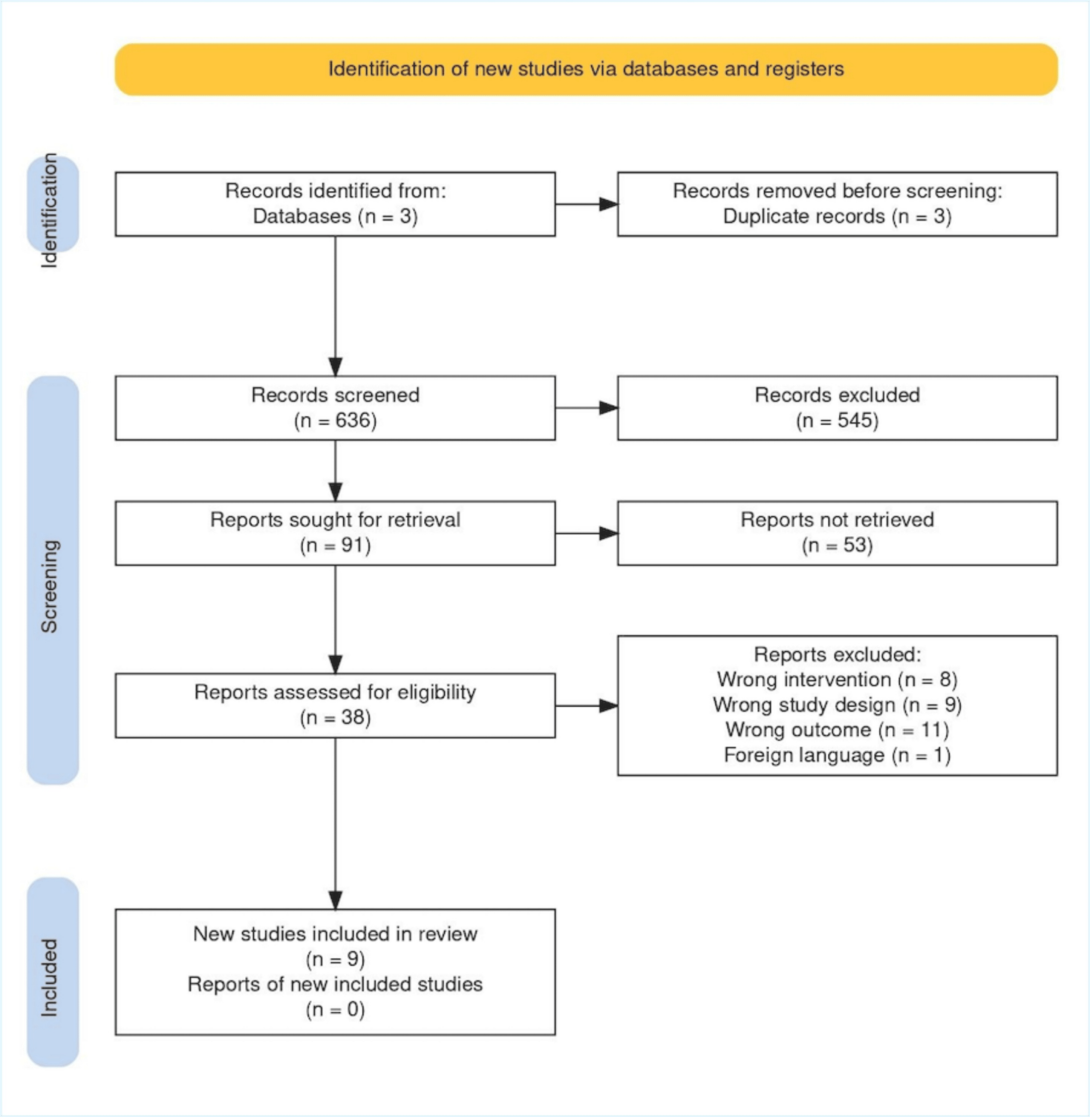
Preferred Reporting Items for Systematic Reviews and Meta-Analyses (PRISMA). Note: 636 records were screened with Preferred Reporting Items for Systematic Reviews and Meta-Analyses (PRISMA) [13]. Records were identified, screened, excluded, sought for retrieval, assessed for eligibility, and excluded for the following reasons: 8 articles had the wrong intervention, 9 studies had the incorrect study design, 11 articles had the incorrect outcome, and 1 was in a foreign language.

Outlined in the PRISMA chart. A total of 636 studies were retrieved, and 38 of these underwent full-text analysis. Ultimately, nine articles were deemed eligible after removing duplicates, screening titles and abstracts, and excluding non-accessible information, as illustrated in Figure 1.

Of all the articles, eight were retrospective cohort studies, and one was a randomized controlled trial. The studies were conducted in China (33%), the USA (22%), the UK (11%), Taiwan (11%), Jordan (11%), and France (11%). The total study population consists of 13,159 patients, of which 2487 underwent MIPD and 10672 underwent OPD. A total of 13,159 participants were included across the studies, with individual study populations ranging from 33 to 11845. Both females and males were assessed. The mean age was 63.58, with a follow-up period of 37 months.

**Figure 2:**
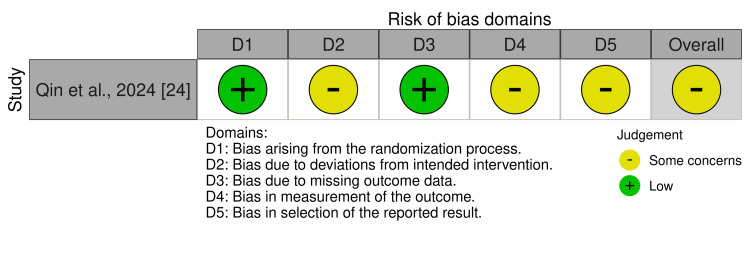
ROB2 tool for assessing the risk of bias in the RCTs. Note: One study was evaluated using the ROB2 tool. The ROB2 tool includes five domains (D1-D5), each assessing different criteria in the study’s methodology. The results were represented as Low Risk, Some Concerns, and High Risk depending on the quality of the methodology [14]. ROB: risk of bias

For the risk of bias assessment, studies were categorized into two groups: RCTs and cohort studies. The RCTs were evaluated using the ROB2 tool, which is freely available and open access through the Cochrane Collaboration [14]. Cohort studies were assessed using the NOS, a widely used tool that is publicly available for research purposes but is not formally published under an open access license [15]. Our selection included a total of nine articles. Among the RCTs, we identified one study, which was rated as “Some concerns” (100%). For the cohort studies, eight studies were evaluated, of which seven were rated as “good quality” (87.5%) and one as “fair quality” (12.5%). The details were presented in Figure 2 and Table 1, along with the criteria assessed for each study.

**Table 1:** Newcastle- Ottawa Tool for Cohort studies’ risk of bias appraisal. Note: Eight studies were evaluated using the Newcastle-Ottawa Quality Assessment Scale (NOS) [15]. The results were represented as Good quality, Fair quality, and Poor quality. The criteria used for each are as follows: Good quality studies: 3 or 4 stars in the selection domain, 1 or 2 stars in the comparability domain, and 2 or 3 stars in the outcome/exposure domain. Fair quality studies: 2 stars in the selection domain, 1 or 2 stars in the comparability domain, and 2 or 3 stars in the outcome/exposure domain. Poor quality studies: 0 or 1 star in the selection domain, 0 stars in the comparability domain, and 0 or 1 star in the outcome/exposure domain.

Author, Year	Study Design	Selection	Comparability	Outcome/ Exposure	Total	Subjective Evaluation
Mungo et al, 2023 [16]	Cohort	4	1	2	7	Good quality
Villano et al, 2023 [17]	Cohort	4	2	2	8	Good quality
Li et al, 2023 [18]	Cohort	4	2	2	8	Good quality
Lai et al, 2012 [19]	Cohort	3	1	2	6	Fair quality
Gall et al, 2020 [20]	Cohort	4	1	3	8	Good quality
Shyr et al, 2024 [21]	Cohort	4	2	3	9	Good quality
Dokmak et al, 2015 [22]	Cohort	4	2	2	8	Good quality
Ammori et al, 2020 [23]	Cohort	4	2	1	7	Good quality

Results of Individual Studies

Lai et al. (2012) performed a total of 87 surgeries. The robotic procedure was successfully carried out in 20 patients. Open surgery was performed on the rest of the patients. Approximately 70% of all the cases had a malignant pathological report. The operative time was 227 minutes longer with minimal invasion. However, blood loss and hospital stay favored the non-traditional approach, with 527 ml less blood loss and 12 fewer days in the hospital, respectively. In total, 10 controls (50%) and 33 cases (49%) experienced postoperative complications. Only 5% (n=1) of cases had to be converted to an open procedure. Two patients suffered postoperative deaths. However, the study did not conclude whether robotic surgery surpasses open surgery [19].

Dokmak et al. (2015) concluded that LPD must be reserved for patients with a low risk of pancreatic fistulae. Forty-one patients developed such complications, and only one patient from the LPD group died as a result. The surgery lasted longer in the LPD group (342 vs. 264 minutes, p < 0.001), with higher bleeding rates (24% vs. 7%, p = 0.02). As highlighted by the authors, postoperative complications decreased as the learning curve advanced. However, they concluded that LPD is not recommended for all patients [22].

A retrospective cohort study published by Ammori et al. (2020) established that “there are potential advantages to laparoscopy when performed by an experienced laparoscopic and pancreatic surgeon”. LPD intervention doubled the surgical time, with an average of 618 minutes. Blood loss was relatively the same in both groups, ranging from 200 to 325 ml. Clavien-Dindo complications greater than III had a p-value of 0.643. Hospital stay was almost 3 days longer in the open surgery group, with an average of 7.8 days (p < 0.0001). It was concluded that only expert hands should execute minimally invasive surgery [23].

Gall et al. (2020) shared their experience with robotic Whipple procedures in the UK, reporting that RPD (n = 25) had longer median operating times (461 minutes) compared to LPD (n = 41) (330 minutes) and OPD (n = 37) (300-350 minutes), with a statistically significant difference (p < 0.0001). Estimated blood loss and transfusion requirements were lower after RPD and LPD compared to OPD (p = 0.012 and p < 0.0001, respectively). No RPD cases required conversion to open surgery, in contrast to 24.4% of LPD cases. Morbidity was comparable across groups, with a Clavien-Dindo score of three observed in 20.00%, 24.39%, and 18.92% of RPD, LPD, and OPD cases, respectively (p = 0.83). Postoperative pancreatic fistula rates were observed in 16.00%, 29.27%, and 21.62% of the RPD, LPD, and OPD cohorts, respectively (p = 0.81). 90-day mortality was reported in 0.97% of the total cohort. The authors concluded that robotic interventions are not inferior to open surgery concerning postoperative and oncological outcomes [20].

Between January 2016 and December 2019, Li et al. evaluated patients at Ruijin Hospital, including those with a BMI > 25 kg/m². Seventy-five patients in the OPD group and 75 in the RPD group were studied. The RPD group demonstrated advantages in terms of estimated blood loss (323.3 mL vs. 480.7 mL, p = 0.010), postoperative abdominal infection rate (24% vs. 44%, p = 0.010), and the incidence of Clavien-Dindo III-V complications (14.7% vs. 28.0%, p = 0.042) compared to the OPD group. In this propensity-matched cohort, they concluded that minimally invasive surgery is safe even for overweight and obese patients [18].

In 2023, Villano et al. published a retrospective cohort study that examined patients who underwent pancreatoduodenectomy. Of these, 80.6% underwent OPD, 19.4% underwent MIPD, and 24% underwent conversion to open. The median overall survival was poorer after conversion to open (21.8 months) compared to OPD (23.6 months) or MIPD (25.2 months). Survival rates significantly worsened after conversion to open surgery. Surgical training and simulation were identified as crucial factors influencing surgical outcomes [17].

Excluding cancer diagnoses, Mungo et al. published a study in Surgical Endoscopy that analyzed data from 188 patients between 2010 and 2020. Of these, 68 patients underwent OPD, and the remaining patients underwent RPD. The postoperative benefits of RPD included a significantly lower incidence of operative pancreatic fistula (10 (8.3%) vs. 24 (35.3%), p < 0.001), fewer surgical site infections (SSI) (9 (7.5%) vs. 11 (16.2%), p = 0.024), shorter operative times, a higher lymph node yield (29, p = 0.001), and lower 90-day mortality (1 (0.8%) vs. 4 (5.9%), p = 0.039). Long-term complications were similar between groups, except for a higher incidence of small bowel obstruction (SBO) (2 (1.7%) vs. 4 (5.9%), p = 0.031) and the need for surgical intervention for SBO (0 (0.0%) vs. 2 (2.9%), p = 0.019) in the OPD group. As a result, the authors encourage the use of RPD for benign or premalignant diseases [16].

In 2024, Qin et al. reviewed a multicenter randomized controlled trial, the TJDBPS01, with a 3-year follow-up. The study included 656 patients, and the overall survival rates were 59.1% for the LPD group and 54.3% for the OPD group (p = 0.33, hazard ratio: 1.16, 95% CI: 0.86-1.56). The 3-year overall survival rates for benign diagnoses were 81.3% in the LPD group and 85.6% in the OPD group (p = 0.40, hazard ratio: 0.70, 95% CI: 0.30-1.63). No significant differences were observed in quality of life, depression, or other outcomes between the two groups. Consistent with the initial study, LPD performed by experienced surgeons yielded results comparable to OPD [24].

In 2023, Shyr et al. published a retrospective cohort study that examined 147 patients with ampullary cancer who underwent pancreaticoduodenectomy. Of these, 101 patients underwent RPD and 46 underwent OPD. Propensity score-matching was applied in a 2:1 ratio, resulting in the analysis of 88 patients in the RPD group and 44 in the OPD group. The operative time was similar between the groups before (median: 6.8 vs. 6.5 hours, p = 0.125) and after (6.5 vs. 6.5 hours, p = 0.283) propensity score-matching. Intraoperative blood loss was lower in the RPD group, both before (median: 120 vs. 320 mL, p < 0.001) and after (100 vs. 335 mL, p < 0.001) propensity score-matching (PSM). No significant differences were found in surgical complications, operative mortality, surgical morbidity, or complication rates according to the Clavien-Dindo classification between RPD and OPD, both before and after PSM. In addition to perioperative outcomes, Shyr et al. also analyzed long-term oncological outcomes. They found no significant differences between the RPD and OPD groups regarding overall survival and disease-free survival, indicating comparable long-term cancer control between the two surgical approaches [21]. Additional data supporting the finding described above are presented in Tables 2-4, labeled as Part 1 of 3, Part 2 of 3, and Part 3 of 3.

**Table 2:** General Outcomes (Part 1 of 3). UK: United Kingdom; LPD: laparoscopic pancreatoduodenectomy; RPD: robotic pancreatoduodenectomy; OPD: open pancreatoduodenectomy; N/A: not available; BMI: body mass index; DM: diabetes mellites

Author, year	Li et al. 2023 [18]	Lai et al. 2012 [19]	Gall et al. 2020 [20]
Title	Short-term outcomes between robot-assisted and open pancreaticoduodenectomy in patients with high body mass index: A propensity score matched study	Robot-assisted laparoscopic pancreaticoduodenectomy versus open pancreaticoduodenectomy: A comparative study	Transition from open and laparoscopic to robotic pancreatoduodenectomy in a UK tertiary referral hepatobiliary and pancreatic center - Early experience of robotic pancreatoduodenectomy
Country	China	China	UK
Study design	Retrospective cohort	Retrospective cohort	Retrospective cohort
Population	150	87	103
Total age, mean	61.6	64.2	62.78
Cases age, mean (SD)	61.8 (10.3)	66.4 (11.9)	60.93 (12.52)
Controls age, mean (SD)	61.5 (9.6)	62.1 (11.2)	63.71 (11.06)
Sex	Both	Both	Both
Intervention	RPD	RPD	RPD
Comparator	OPD	OPD	LPD and OPD
Comorbidities	Overweight, obesity, DM, history of smoking/alcohol	N/A	N/A
Follow-up time (months)	N/A	N/A	22
No. of cases	75	20	25
No. of controls	75	67	78
Clavien-Dindo classification ≥ III cases	N/A	N/A	5
Clavien-Dindo classification ≥ III control	N/A	N/A	17
Blood transfusion cases (IQR)	N/A	N/A	0
Blood transfusion controls (IQR)	N/A	N/A	8
Duct diameter < 3 mm Cases	N/A	N/A	N/A
Duct diameter > 3 mm Cases	N/A	N/A	N/A
Duct diameter < 3 mm Controls	N/A	N/A	N/A
Duct diameter > 3 mm Controls	N/A	N/A	N/A
Time of surgery, minutes cases	301.3	491	461
Time of surgery, minutes controls	303.3	264.9	330
Complication cases	18	10	N/A
Complication controls	33	33	N/A
Mortality cases	1	0	1
Mortality controls	6	2	0
Key points	The study compares short-term outcomes between robot-assisted and OPD in high BMI patients, showing that RPD results in lower blood loss, fewer severe complications, reduced abdominal infections, and lower 90-day mortality, confirming it as a safer and effective option.	RPD had a longer operative time than OPD. RPD showed reduced blood loss and shorter hospital stay. No significant difference in mortality rates, R0 resection rates, or lymph node harvest. Robotic PD was feasible but requires further validation for long-term outcomes.	The study aims to make a retrospective review of a prospectively collected database of the first consecutive cases of RPD, LPD, and OPD. Of 103 patients, 25 were part of the case group, 78 formed the control group, 37 were divided into OPD, and 41 into LPD. RPD included more operative time and mortality during the 22 months following. Nevertheless, there were fewer postoperative complications and blood transfusion requirements after RPD.

**Table 3:** General Outcomes (Part 2 of 3). Note: General outcomes summarize the characteristics of the nine included studies. The majority are cohort studies (n=8), with the remainder being RCTs (n=1). The range of participants varies from 33 to 11845, encompassing multiple geographic regions. The table includes the characteristics of each intervention group, the type of intervention performed, as well as the parameters used to assess the efficacy and safety of each procedure. RCTs: randomized controlled trials; LPD: laparoscopic pancreatoduodenectomy; RPD: robotic pancreatoduodenectomy; OPD: open pancreatoduodenectomy; N/A: Not available; DM: diabetes mellites; HTN: hypertension

Author, year	Shyr et al. 2024 [21]	Dokmak et al. 2015 [22]	Ammori et al. 2020 [23]
Title	Survival and surgical outcomes of robotic versus open pancreatoduodenectomy for ampullary cancer: A propensity score-matching comparison	Laparoscopic pancreaticoduodenectomy should not be routine for resection of periampullary tumor	A case-matched comparative study of laparoscopic versus open pancreaticoduodenectomy
Country	Taiwan	France	Jordan
Study design	Retrospective cohort	Retrospective cohort	Retrospective cohort
Population	132	92	33
Total age, mean	N/A	N/A	N/A
Cases age, mean (SD)	67 (43-95)	60 (27-85)	57 (48-72)
Controls age, mean (SD)	65 (44-87)	63 (47-81)	63 (22-75)
Sex	Both	Both	Both
Intervention	RPD	LPD	LPD
Comparator	OPD	OPD	OPD
Comorbidities	N/A	DM, HTN	N/A
Follow-up time (months)	N/A	N/A	90
No. of cases	88	46	11
No. of controls	44	46	22
Clavien-Dindo classification ≥ III cases	15	13	1
Clavien-Dindo classification ≥ III control	5	9	1
Blood transfusion cases (IQR)	N/A	5	0
Blood transfusion controls (IQR)	N/A	4	0
Duct diameter < 3 mm Cases	42	N/A	6
Duct diameter > 3 mm Cases	42	N/A	6
Duct diameter < 3 mm Controls	26	N/A	10
Duct diameter > 3 mm Controls	18	N/A	12
Time of surgery, minutes Cases	432	342	680
Time of surgery, minutes Controls	402	264	313
Complication cases	N/A	34	4
Complication controls	N/A	27	13
Mortality cases	0	1	0
Mortality controls	0	0	0
Key points	This study aimed to clarify the feasibility and justification of robotic pancreaticoduodenectomy in ampullary cancer in terms of surgical risks and oncologic and survival outcomes. The operation time showed no significant difference after matching. There were no significant differences. The survival outcomes were also similar between the two groups, regardless of matching.	The aim was to compare the outcomes of LPD and OPD. Lower BMI and a soft pancreas were observed in patients with LPD, but there were no differences in associated comorbidities or underlying disease. Surgery lasted longer in the LPD group. One death occurred in the LPD group, and severe morbidity was higher in LPD due to grade C pancreatic fistula (PF), bleeding, and revision surgery. Pathologic examination for malignant diseases did not identify any differences.	The aim of this study was to compare the outcomes of these two approaches at a tertiary cancer center in the Middle East. The groups were comparable for age and sex distribution, tumor size (3 cm in each group), frequency of pancreatic duct dilatation, and malignant pathology. Although the operating time for LPD was significantly longer, LPD was associated with significantly shorter primary and total hospital stays that included readmissions. There were no significant differences. In patients with malignant disease, there were no differences with regard to the number of lymph nodes retrieved and the frequency of R0 resections.

**Table 4:** General Outcomes (Part 3 of 3). USA: United States of America; RCTs: randomized controlled trials; LPD: laparoscopic pancreatoduodenectomy; RPD: robotic pancreatoduodenectomy; OPD: open pancreatoduodenectomy; N/A: Not available; MIPD: minimally invasive pancreaticoduodenectomy

Author, year	Qin et al. 2024 [24]	Mungo et al. 2023 [16]	Villano et al. 2023 [17]
Title	Effect of laparoscopic and open pancreaticoduodenectomy for pancreatic or periampullary tumors	Pancreaticoduodenectomy for benign and premalignant pancreatic and ampullary disease is a robotic surgery, a better approach	Discrepancies in survival after conversion to open in minimally invasive pancreatoduodenectomy
Country	China	USA	USA
Study design	RCT	Retrospective cohort	Retrospective cohort
Population	529	188	11845
Total age, mean	58.5	68 (57.73)	66.4
Cases age, mean (SD)	59.2 (10.2)	67 (57.73)	66.7 (10.1)
Controls age, mean (SD)	57.8 (10.8)	68 (59.74)	66.4 (10.2)
Sex	Male	Female	Both
Intervention	LPD	RPD	LPD
Comparator	OPD	OPD	OPD and conversions to open surgery
Comorbidities	N/A	HTN, DM, and coronary artery disease	Charlson comorbidity index
Follow-up time (months)	36	3	N/A
No. of cases	268	120	1834
No. of controls	261	68	10011
Clavien-Dindo classification ≥ III Cases	76	15	N/A
Clavien-Dindo classification ≥ III Control	58	7	N/A
Blood transfusion cases (IQR)	60	N/A	N/A
Blood transfusion controls (IQR)	79	N/A	N/A
Duct diameter < 3 mm Cases	161	3	N/A
Duct diameter > 3 mm Cases	0	0	N/A
Duct diameter < 3 mm Controls	150	3.5	N/A
Duct diameter > 3 mm Controls	0	0	N/A
Time of surgery, minutes Cases	320	353	391
Time of surgery, minutes Controls	300	456.6	447
Complication cases	132	10	N/A
Complication controls	116	24	N/A
Mortality cases	5	1	92
Mortality controls	6	4	647
Key points	The objective of this study was to estimate whether the potential short-term advantages of LPD could allow patients to recover in a timelier manner and achieve better long-term survival than with OPD in patients with pancreatic or periampullary tumors. Data from 656 male patients who underwent pancreaticoduodenectomy were analyzed. Finally, 529 patients were included in the analysis, including 2658 patients in the LPD group and 261 in the OPD group. For malignancies, the 3-year overall survival rates were 59.1% for LPD group and 54.3% for OPD group.	This study aims to compare peri-operative and long-term outcomes of OPD and RPD. 188 patients met the inclusion criteria, of which 68 were OPD and 120 RPD. Postoperative merits of the RPD included lower clinically relevant postoperative pancreatic fistula 10 (8.3%) vs 24 (35.3%), fewer surgical sites of infections, shorter operative time, greater lymph node yield, and lower 90-day mortality; 1 (0.8%) vs 4 (5.9%).	The objective of this study is to compare survival rates in patients undergoing MIPD who successfully completed the procedure versus those who required conversion to open surgery. The main findings indicate that patients who underwent MIPD and successfully completed the procedure had better survival outcomes compared to those who required conversion to open surgery.

Discussion

The pancreas is a retroperitoneal organ in the human body with both exocrine and endocrine functions. Pancreatic tumors can be classified into two groups: non-endocrine pancreatic tumors and endocrine pancreatic tumors. Non-endocrine pancreatic tumors can be either benign or malignant. Benign non-endocrine pancreatic tumors include adenoma, cystadenoma, lipoma, fibroma, hemangioma, lymphangioma, and neuroma. Malignant pancreatic tumors exhibit a variety of histological characteristics, including pancreatic ductal adenocarcinoma, sarcoma, and metastatic tumors [1]. Approximately 90% of pancreatic cancers are PDAC [2].

Pancreatoduodenectomy is one of the most challenging abdominal surgeries and remains the standard treatment for pancreatic and periampullary tumors [24]. Minimally invasive surgery offers several advantages over open techniques, including reduced pain, decreased blood loss, and a faster return to functional activities [20]. Despite these advantages, the primary goal of this study was to compare both MIPD and OPD, focusing on the outcomes of each technique. This includes examining the incidence of complications as well as the duration of complete recovery.

Multiple studies from various geographic regions were retrieved, comprising a total of 13,159 patients, with study populations ranging from 33 to 11845. Qin et al. and Villano et al. established that LPD not only offers technical feasibility and safety but also advantages such as reduced hospital stays and faster recovery [17, 24]. Similarly, Mungo et al. analyzed data from 188 patients, determining that RPD is both relevant and safe for patients with benign and premalignant pancreatic and ampullary disease [16]. Li et al. reviewed patients with high body mass index, a recognized risk factor for minimally invasive surgery, acknowledging that MIPD is safe even for overweight and obese patients [18].

Lai et al. performed 20 successful RPD procedures and 67 OPD, concluding that blood loss and hospital stay favored the robotic technique. However, the study did not determine whether robotic surgery was superior to open surgery [19]. Dokmak et al. determined that LPD should be reserved for patients with a low risk of pancreatic fistulae. One patient in the laparoscopic group in his study died because of such a complication, concluding that LPD is not recommended for all patients [22]. Ammori et al. deduced that laparoscopic intervention doubled the surgical time compared to open intervention [23]. Gall et al. conducted a study in which 25 patients underwent RPD, compared to 41 patients who underwent LPD and 37 who underwent OPD. They established that robotic interventions were not inferior to open surgery, a conclusion supported by Shyr et al. [20,21].

The studies included in this systematic review provided a comprehensive evaluation of the efficacy of MIPD compared to OPD in patients with pancreatic ductal adenocarcinoma. The primary objective of our study was to compare the treatment outcomes of MIPD with OPD, focusing on postoperative outcomes, blood loss, and the reduction in hospital stays. Nine studies were included, involving both male and female patients over the age of 18. Four of the nine studies concluded that MIPD offered technical feasibility and safety, along with reduced hospital stays and faster recovery. In contrast, the remaining five studies found that MIPD was not superior to OPD, although it was associated with less blood loss and shorter hospital stays. Other authors emphasized that MIPD should be reserved for patients with a low risk of pancreatic fistulae and that the procedure should be performed only in carefully selected patients.

Despite the benefits offered by MIPD, a debate continued regarding the safety and advantages of using the laparoscopic approach for this complex resection and reconstruction procedure. Complications such as pancreatic fistulae, extended surgical time, and other factors did not favor MIPD. In terms of complications, we analyzed the incidence of those classified as Clavien-Dindo grade III to V in the MIPD groups. Qin et al. reported that 132 patients who underwent LPD developed complications, compared to 116 patients in the OPD group [24].

Upon examining the mortality rates across all studies, no substantial improvement was noted in comparison to OPD. Most studies reported a lower 30-day mortality rate. Lai et al. mentioned two postoperative deaths due to complications. However, the operating time is generally shorter with minimally invasive techniques [19]. Ammori et al. reported that the surgical time for LPD was double that of OPD. Apart from Ammori’s report, no other author noted a significant increase in surgical time. Regarding hospitalizations, MIPD demonstrated fewer days of hospitalization [23].

Considering the limitations of the study, such as the lack of RCTs comparing MIPD with OPD, further research with an expanded patient database is needed to address the discrepancies in postoperative complications and surgical time between the two procedures. Additionally, a larger body of RCTs would help clarify the differences among the authors’ conclusions regarding the use of MIPD as a treatment for pancreatic ductal adenocarcinoma.

Furthermore, most included studies primarily reported perioperative outcomes, with limited information on long-term oncologic results such as recurrence rates, disease-free survival, and margin status. This highlights a gap in the literature and underscores the need for future research to emphasize these critical long-term cancer outcomes to better inform surgical decision-making.

Lastly, the included studies involved a heterogeneous patient population, such as differences in disease type (benign versus malignant) and BMI. This variability may influence the comparability of results and limit the ability to draw conclusions across studies.

Conducting more randomized controlled trials and expanding the patient database would significantly influence decision-making regarding minimally invasive procedures as the preferred first-line treatment. These procedures should be performed by experienced surgeons proficient in minimally invasive techniques.

## Conclusions

In our systematic review, MIPD offers several benefits. These include shorter hospital stays, faster recovery, lower estimated blood loss, and higher surgical viability. However, it also presents challenges, such as prolonged surgical time and a higher incidence of postoperative complications, such as pancreatic fistula. To achieve the best benefits with this technique, it should be performed by an experienced surgeon. This suggests that minimally invasive techniques for the treatment of pancreatic cancer are viable and safe. Nonetheless, further RCTs with low risk of bias are needed to make the best clinical decisions with the best available evidence.

In conclusion, the findings of our systematic review indicate that these techniques may represent a viable option for managing pancreatic cancer. Nevertheless, given the limited RCTs, their application should be restricted to cases that are appropriately selected.
